# OP68 Adaptation Of Processes For HTA Of Digital Health Technologies Based On Artificial Intelligence

**DOI:** 10.1017/S0266462324001272

**Published:** 2025-01-07

**Authors:** Carolina Moltó-Puigmartí, Joan Segur-Ferrer, Didier Domínguez Herrera, Susanna Aussó Trias, Rosa Maria Vivanco-Hidalgo

## Abstract

**Introduction:**

The advent of artificial intelligence (AI) in digital health technologies (DHT) requires a comprehensive health technology assessment (HTA) to ensure safety and effectiveness and to demonstrate the value of these technologies in healthcare systems. Recognizing the unique requirements posed by AI-based DHT, our agency has undertaken several initiatives to tailor and adapt our processes for effective HTA.

**Methods:**

We started by identifying the processes that were not working optimally and planned a list of actions needed to improve them. These actions were: (i) to develop a new evaluation framework for the assessment of DHT, including those based on AI; (ii) to increase our activity on early HTA; (iii) to seek collaboration with an organization for technical assessment of AI, with a particular emphasis on trustworthy AI requirements; (iv) to adapt our HTA report templates; (v) to create new forms to request information from the technology developers; and (vi) to set up a working group on HTA of AI-based DHT.

**Results:**

We have now an evaluation framework that informs on the relevant aspects for HTA of AI-based DHT and the evidence that developers need to generate in order to proof the value of their technology. We designed a circuit to identify promising technologies and increased our early HTA work for timely advice. The evaluation team now involves an additional partner for the technical assessment domain. In addition, we have new templates for early HTA reports, which explain those AI-specific elements to be addressed, as well as industry information request forms that enable collecting specific information like algorithm type and population used for clinical validation.

**Conclusions:**

Tailoring HTA processes to AI-based DHT is crucial in today’s fast-paced health technology landscape. Our new evaluation framework, the involvement of new partners in the assessment team, the creation of new templates, and enhanced early HTA work helps to evaluate these technologies optimally. We are also setting up a working group to ensure homogeneous evaluation within Spain.

